# Supplementary material for: Scavenging of Superoxide in Aprotic Solvents of Four Isoflavones That Mimic Superoxide Dismutase
Source: Int J Mol Sci. 2023 Feb 14;24(4):3815. doi: 10.3390/ijms24043815 (PMC9965188; doi:10.3390/ijms24043815)
Supplement: Supplementary file 1 [file ijms-24-03815-s001.zip › ijms-2168849-supplementary.pdf]

Video S1. The π-π interaction between Biochanin-A ring C and superoxide acts like sigma scavenging on H(hydroxyl) of ring A

Biochanin_ringC_vd
W_piO2Rad_GoesSI

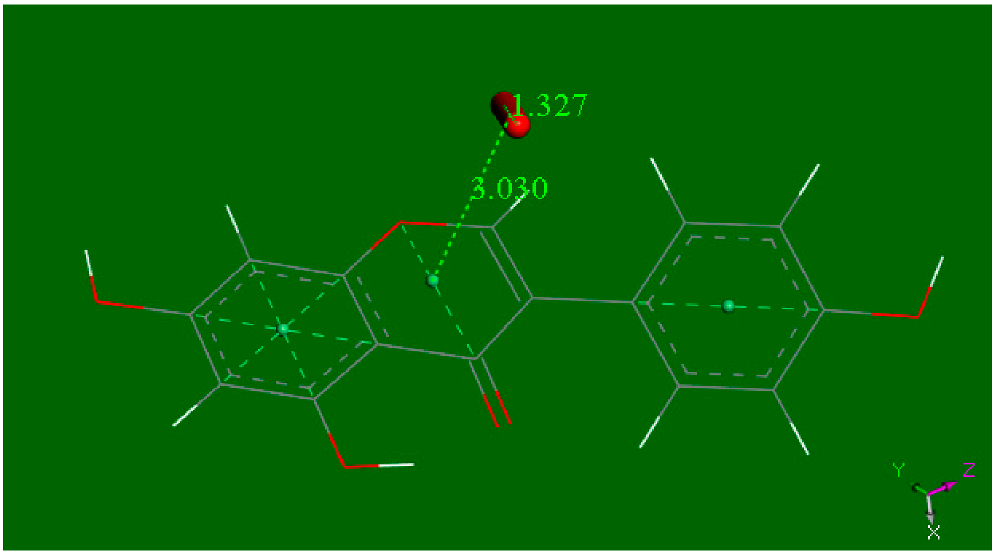

Figure S2. DFT minimization of superoxide π–π posed over genistein ring C forms a superoxide-η-genistein complex.

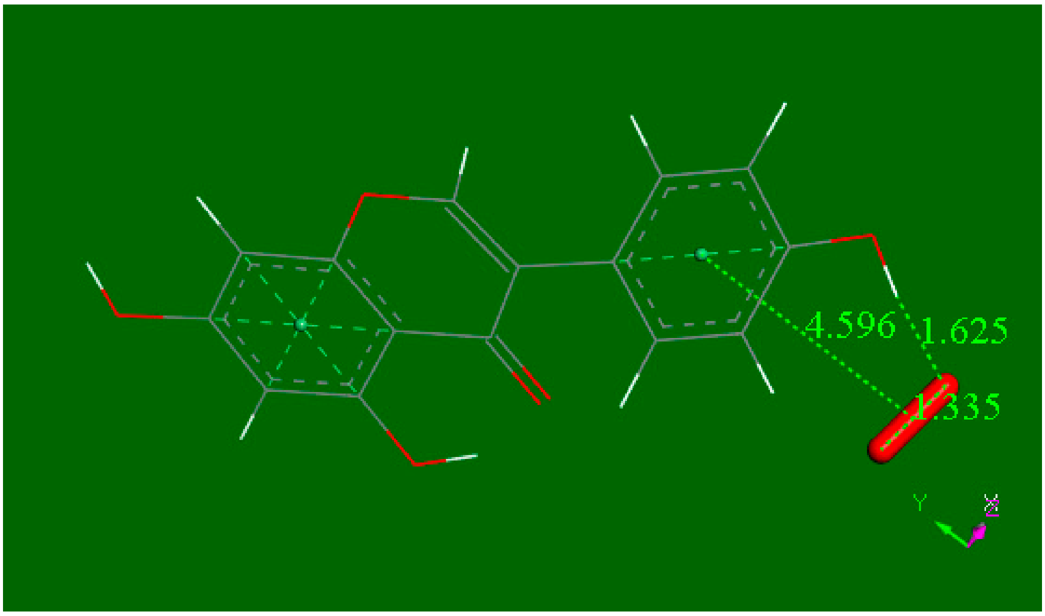

Figure S3. DFT minimization of superoxide π–π posed over genistein ring B forms a H-bond with H4′(hydroxyl).

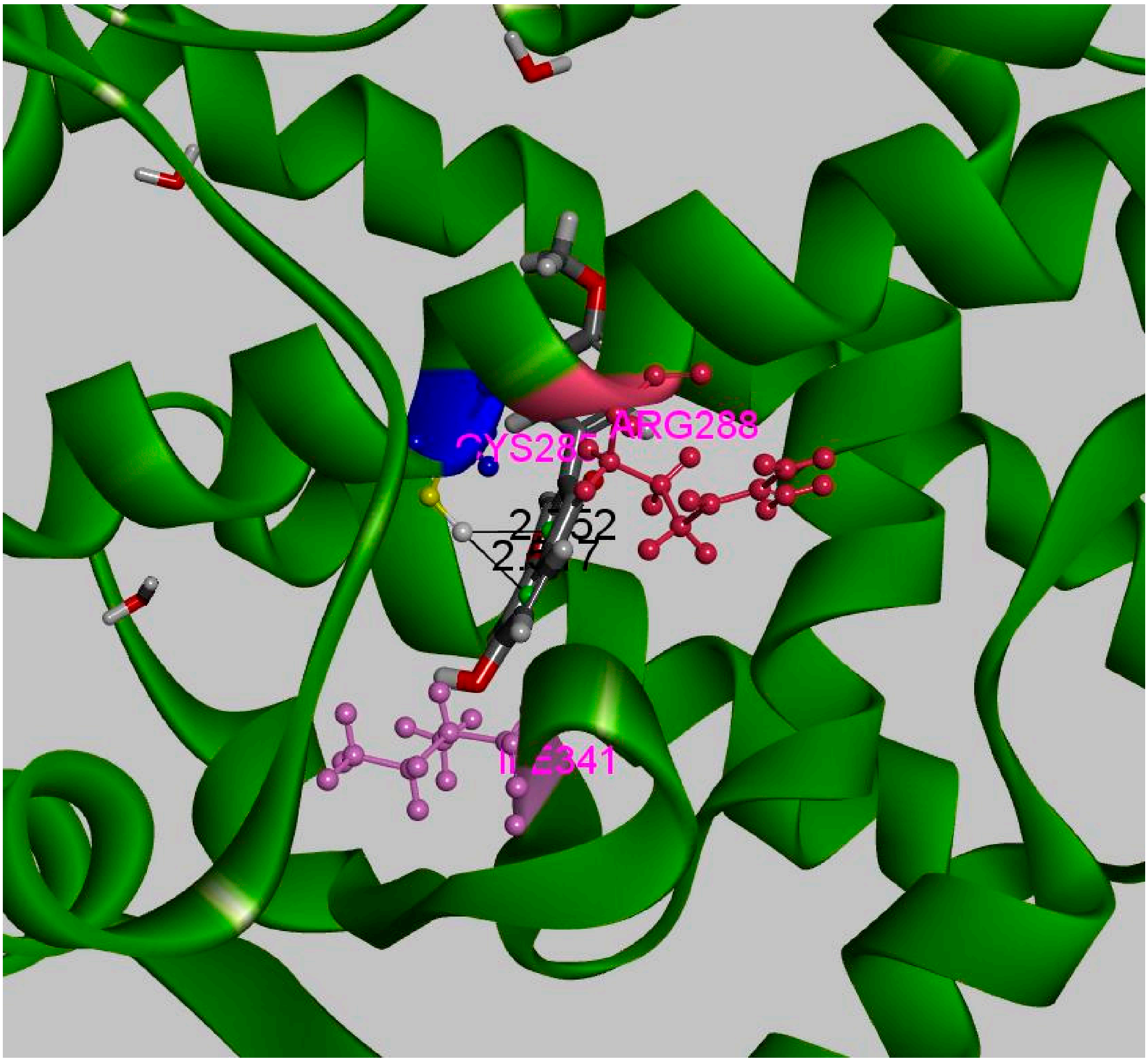

Figure S4. Calculating binding energy (−22.1 Kcal/mol) for a partial view of 5UGM protein including FMNT pose 5 (stick style) after dynamic cascade. Amino acid Cys285 (blue) has its S in yellow and its associated H atom in white. Arg288 (copper) and Ile341 (cyan) are also displayed as a ball style. H(Cys285) separation from ring centroids is 2.517 Å (ring A) and 2.572 Å (pyrone ring).
